# *CerebNet*: A fast and reliable deep-learning pipeline for detailed cerebellum sub-segmentation

**DOI:** 10.1016/j.neuroimage.2022.119703

**Published:** 2022-12-01

**Authors:** Jennifer Faber, David Kügler, Emad Bahrami, Lea-Sophie Heinz, Dagmar Timmann, Thomas M. Ernst, Katerina Deike-Hofmann, Thomas Klockgether, Bart van de Warrenburg, Judith van Gaalen, Kathrin Reetz, Sandro Romanzetti, Gulin Oz, James M. Joers, Jorn Diedrichsen, ESMI MRI Study Group, Paola Giunti, Hector Garcia-Moreno, Heike Jacobi, Johann Jende, Jeroen de Vries, Michal Povazan, Peter B. Barker, Katherina Marie Steiner, Janna Krahe, Martin Reuter

**Affiliations:** 1 Ataxia Centre, Department of Clinical and Movement Neurosciences, UCL Queen Square Institute of Neurology & National Hospital for Neurology and Neurosurgery, University College London Hospitals NHS Foundation Trust, London, UK; 3 Department of Neurology, University Hospital of Heidelberg, Heidelberg, Germany; 4 Department of Neuroradiology, Heidelberg University Hospital, Heidelberg, Germany; 5 Department of Neurology, Expertise Center Movement Disorders Groningen, University Medical Center Groningen, University of Groningen, The Netherlands; 6 Johns Hopkins University School of Medicine, Baltimore, MD, U.S.; 8 Department of Neurology, Center for Translational Neuro, and Behavioral Sciences (C-TNBS), University Hospital Essen, University of Duisburg-Essen, Essen, Germany; 9 Department of Neurology, RWTH Aachen University, Germany; a German Center for Neurodegenerative Diseases (DZNE), Bonn, Germany; b Computer Science Department, University Bonn, Bonn, Germany; c Department of Neurology, University Hospital Bonn, Germany; d Department of Neurology, Center for Translational Neuro, and Behavioral Sciences (C-TNBS), University Hospital Essen, University of Duisburg-Essen, Essen, Germany; e Department of Neuroradiology, University Hospital Bonn, Germany; f Department of Neurology, Donders Institute for Brain, Cognition, and Behaviour, Radboud university medical center, Nijmegen, The Netherlands; g Department of Neurology, RWTH Aachen University, Germany; h JARA-Brain Institute Molecular Neuroscience and Neuroimaging, Forschungszentrum Jülich, Germany; i Center for Magnetic Resonance Research, Department of Radiology, University of Minnesota, Minneapolis, MN, USA; j Departments of Computer Science and Statistical and Actuarial Sciences, Western University, London, ON, Canada; k A.A. Martinos Center for Biomedical Imaging, Massachusetts General Hospital, Boston, MA, USA; l Department of Radiology, Harvard Medical School, Boston, MA, USA

**Keywords:** CerebNet, Cerebellum, Computational neuroimaging, Deep learning, 00-01, 99-00

## Abstract

Quantifying the volume of the cerebellum and its lobes is of profound interest in various neurodegenerative and acquired diseases. Especially for the most common spinocerebellar ataxias (SCA), for which the first antisense oligonculeotide-base gene silencing trial has recently started, there is an urgent need for quantitative, sensitive imaging markers at pre-symptomatic stages for stratification and treatment assessment. This work introduces *CerebNet*, a fully automated, extensively validated, deep learning method for the lobular segmentation of the cerebellum, including the separation of gray and white matter. For training, validation, and testing, T1-weighted images from 30 participants were manually annotated into cerebellar lobules and vermal sub-segments, as well as cerebellar white matter. *CerebNet* combines *FastSurferCNN*, a UNet-based 2.5D segmentation network, with extensive data augmentation, e.g. realistic non-linear deformations to increase the anatomical variety, eliminating additional preprocessing steps, such as spatial normalization or bias field correction. *CerebNet* demonstrates a high accuracy (on average 0.87 Dice and 1.742mm Robust Hausdorff Distance across all structures) outperforming state-of-the-art approaches. Furthermore, it shows high test-retest reliability (average ICC >0.97 on OASIS and Kirby) as well as high sensitivity to disease effects, including the pre-ataxic stage of spinocerebellar ataxia type 3 (SCA3). *CerebNet* is compatible with *FreeSurfer* and *FastSurfer* and can analyze a 3D volume within seconds on a consumer GPU in an end-to-end fashion, thus providing an efficient and validated solution for assessing cerebellum sub-structure volumes. We make *CerebNet* available as source-code (https://github.com/Deep-MI/FastSurfer).

## 1 Introduction

For decades, the cerebellum was attributed to have an exclusive role in motor control. Recently, growing evidence suggests a more general involvement of the cerebellum in the adaptive control also of cognitive and emotional processing. In fact, morphometric studies demonstrate significant cerebellar atrophy with age and in a number of non-motor brain diseases, e.g. schizophrenia, autism or Alzheimer’s disease (Diedrichsen, Zotow, Ewa, 2015, D’Mello, Crocetti, Mostofsky, Stoodley, 2015, Han, An, Carass, Prince, Resnick, 2020, Lin, Chen, Tom, Kuo, for the Alzheimer’s Disease Neuroimaging Initiative, 2020, Marek, Siegel, Gordon, Raut, Gratton, Newbold, Ortega, Laumann, Adeyemo, Miller, Zheng, Lopez, Berg, Coalson, Nguyen, Dierker, Van, Hoyt, McDermott, Norris, Shimony, Snyder, Nelson, Barch, Schlaggar, Raichle, Petersen, Greene, Dosenbach, 2018, Okugawa, Sedvall, Agartz, 2003, Toniolo, Serra, Olivito, Marra, Bozzali, Cercignani, 2018, Webb, Sparks, Friedman, Shaw, Giedd, Dawson, Dager, 2009, Womer, Tang, Harms, Bai, Chang, Jiang, Wei, Wang, Barch, 2016). In healthy humans, the representation of cerebral networks and cognitive domains has been investigated using functional connectivity (Buckner et al., 2011) as well as task functional MRI (King et al., 2019). These complementary studies have helped to increase knowledge about the role of the cerebellum in cognitive and emotional processes. This notwithstanding, the cerebellum is crucial for motor control, in particular metric and power of target movements. With regard to movement disorders, cerebellar atrophy is the characterizing feature in ataxias, which manifest as acquired, genetic, or sporadic degenerative diseases. With clinical features including progressive loss of balance, coordination deficits, and slurred speech, ataxia patients suffer substantial restrictions of mobility and communicative skills. In genetic ataxies, such as the worldwide most common autosomal dominantly inherited spinocerebellar ataxia type 3 (SCA3), the manifest or ataxic stage of the disease is preceded by a pre-ataxic stage, in which neurodegeneration is already quantifiable, e.g., as cerebellar atrophy, while manifest ataxia is not yet present (Faber, Schaprian, Berkan, Reetz, França, de Rezende, Hong, Liao, van de Warrenburg, van Gaalen, Durr, Mochel, Giunti, Garcia-Moreno, Schoels, Hengel, Synofzik, Bender, Oz, Joers, de Vries, Kang, Timmann-Braun, Jacobi, Infante, Joules, Romanzetti, Diedrichsen, Schmid, Wolz, Klockgether, 2021, Kim, Kim, Lee, Lee, 2021, Rezende, de Paiva, Martinez, Lopes-Cendes, Pedroso, Barsottini, Cendes, França, 2018). Preventive interventions that aim to silence the disease gene in pre-ataxic mutation carriers offer a promising treatment option prior to clinical onset (McLoughlin et al., 2018). Now, that the first clinical gene silencing trial has recently started (ClinicalTrials.gov Identifier: NCT05160558), there is an urgent need for non-invasive biomarkers to assess disease manifestation and progression, and to quantify potential treatment effects as clinical scales lack sensitivity during the pre-ataxic stage. Accurate cerebellar volume estimation from structural MRI is a relevant neuroanatomical marker. However, fast automated determination of cerebellar volumes is required, as detailed, manual volumetry, especially of sub-regions, is too time-consuming. Clearly, automated segmentation will benefit various study designs, by reducing workload and by improving reliability.

In the present work, we introduce *CerebNet*, an automated method to sub-segment the cerebellum at the lobular level based on T1-weighted MRI. Our labels focus on a detailed boundary delineation between cerebellar gray matter (CGM) and cerebellar white matter (CWM) capturing the branches of CWM that reach into the cerebellar cortex based on T1-weighted MRI. Our deep learning method leverages the *FastSurfer* approach (Henschel et al., 2020) of multiple 2D networks and minimal pre-processing to obtain detailed boundary segmentations. Since *CerebNet* does not require any preprocessing steps and performs the localization and segmentation of 27 cerebellar regions in only 12 seconds per MRI, it is optimally suited to also efficiently process and screen in large data sets. With very labor-intensive manual reference segmentation, the methodological challenge is to achieve high accuracy and generalizability despite a small reference dataset. To this effect, we perform extensive pre-training on representative cross-study datasets and apply several data augmentation steps including realistic non-linear deformations to ensure wide applicability. Moreover, we validate our method with respect to test-retest reliability and in an association study of neuro-morphometric cerebellum markers across 109 SCA3 mutation carriers, including 42 pre-ataxic participants, as well as 41 healthy controls. Results reveal stronger group differences for *CerebNet* consistent with known patterns of neurodegenerative changes.

### 1.1 Protocols and anatomical reference

The Schmahmann atlas (Schmahmann et al., 1999) is the standard anatomical reference for cerebellar cortex sub-segmentation protocols (Bogovic, Jedynak, Rigg, Du, Landman, Prince, Ying, 2013, Park, Pipitone, Baer, Winterburn, Shah, Chavez, Schira, Lobaugh, Lerch, Voineskos, Chakravarty, 2014) including the “Spatially Unbiased Infratentorial Template” (SUIT) (Diedrichsen, 2006). It introduces a unified terminology of the nomenclature. Slices of the cerebellum are directly compared with the corresponding slices of MR images, thus facilitating the identification of anatomical landmarks. Briefly summarized, the CGM is macroscopically subdivided into the midline vermis and four hemispheric lobes: the anterior, posterior-superior, posterior-inferior, and the flocculonodular lobe. The anterior and posterior lobes are further subdivided into lobules. The vermis is subdivided analogously to the hemispheres except for the anterior lobe. Like all previous protocols, ours follows the nomenclature introduced by Schmahmann (Schmahmann et al., 1999) and our segmentation is largely comparable to previous protocols (Bogovic, Jedynak, Rigg, Du, Landman, Prince, Ying, 2013, Diedrichsen, 2006, Park, Pipitone, Baer, Winterburn, Shah, Chavez, Schira, Lobaugh, Lerch, Voineskos, Chakravarty, 2014). The protocols for segmenting the cerebellum on MR images differ in the level of detail at which single anterior lobules and vermal subsegments are distinguished or aggregated (Bogovic, Jedynak, Rigg, Du, Landman, Prince, Ying, 2013, Diedrichsen, 2006, Park, Pipitone, Baer, Winterburn, Shah, Chavez, Schira, Lobaugh, Lerch, Voineskos, Chakravarty, 2014). Previous work has largely only differed in finding an aggregation compromise in the level of detail for segments I-V. We detail a comparison between the different segmentation protocols as well as to related automated segmentation procedures in the appendix of our protocol for manual segmentation(Heinz et al., 2022). It should be noted, that all previous protocols ignore the CWM strands projecting into the cerebellar cortex (Bogovic, Jedynak, Rigg, Du, Landman, Prince, Ying, 2013, Diedrichsen, 2006, Park, Pipitone, Baer, Winterburn, Shah, Chavez, Schira, Lobaugh, Lerch, Voineskos, Chakravarty, 2014) simplifying the CGM/CWM boundary to a connection line across the base of CWM strands. In consequence, details at the CGM/CWM boundary of the cerebellum are not captured by any of the previous protocols. To allow deeper analysis of the GM/WM boundary in the cerebellum, we extend our protocol by a fine-grained segmentation of CWM strands projecting into the cerebellar cortex. To foster reproducibility and extensibility, we establish and publish our illustrated segmentation protocol online with this publication (Heinz et al., 2022).

### 1.2 Automated methods for cerebellar sub-segmentation

Several methods have been presented for segmenting cerebellar sub-structures including both semi-automated (Pierson et al., 2002) and fully automated (Bogovic, Prince, Bazin, 2013, Carass, Cuzzocreo, Han, Hernandez-Castillo, Rasser, Ganz, Beliveau, Dolz, Ben Ayed, Desrosiers, Thyreau, Romero, Coupé, Manjón, Fonov, Collins, Ying, Onyike, Crocetti, Landman, Mostofsky, Thompson, Prince, 2018, Diedrichsen, 2006, Han, Carass, He, Prince, 2020) approaches. While previous methods relied on atlas-based registration (Diedrichsen, 2006, Diedrichsen, Balsters, Flavell, Cussans, Ramnani, 2009, Park, Pipitone, Baer, Winterburn, Shah, Chavez, Schira, Lobaugh, Lerch, Voineskos, Chakravarty, 2014, Plassard, Yang, Rane, Prince, Claassen, Landman, 2016, Romero, Coupé, Giraud, Ta, Fonov, Park, Chakravarty, Voineskos, Manjón, 2017), artificial neural networks (Powell et al., 2008), support vector machines (Powell et al., 2008), level sets (Bogovic et al., 2013a), active appearance models (Price et al., 2014), and patch matching (Romero, Coupé, Giraud, Ta, Fonov, Park, Chakravarty, Voineskos, Manjón, 2017, Weier, Fonov, Lavoie, Doyon, Louis Collins, 2014), recent work introduced deep learning (Han, Carass, He, Prince, 2020, Han, He, Carass, Ying, Prince, 2019).

The reference method “Spatially Unbiased Infra-tentorial Template” (SUIT) (Diedrichsen, 2006) pioneered fully automatic cerebellum sub-segmentation using non-linear registration to an atlas. Powell et al. (2008) compared atlas registration with fully connected neural network and support vector machine segmentation methods, and demonstrated superior performance of learning approaches. ACCLAIM (Bogovic et al., 2013a), which is based on the Multiple object Geometric Deformable Model framework (Bogovic, Prince, Bazin, 2013, Carass, Prince, 2016), adapts a random forest for boundary classification to produce topologically correct results. The Multiple Automatically Generated Templates brain segmentation algorithm (MAGeT) (Chakravarty, Steadman, van Eede, Calcott, Gu, Shaw, Raznahan, Collins, Lerch, 2013, Park, Pipitone, Baer, Winterburn, Shah, Chavez, Schira, Lobaugh, Lerch, Voineskos, Chakravarty, 2014) creates a template library, then non-linearly registers the target image to each template. The final segmentation is achieved by fusing multiple segmentations using majority voting. The Cerebellar Analysis Toolkit (CATK) (Price et al., 2014) adapts Bayesian active appearance modeling (Patenaude et al., 2011) to generate statistical models for shape and texture and their inter-relationship as priors. RASCAL (Weier et al., 2014) utilizes a patch matching-based approach, which improves the multi-atlas segmentation fusion method of Coupe et al. (Coupé et al., 2011) via majority voting for label fusion and nonlinear registration. CERES (Romero et al., 2017), another patch matching-based segmentation tool, employs the Optimized PatchMatch Label fusion (OPAL) method (Giraud, Ta, Papadakis, Manjón, Collins, Coupé, 2016, Ta, Giraud, Collins, Coupé, 2014). CERES2 (Carass et al., 2018) improves upon CERES (Romero et al., 2017) by adding a patch-based boosted neural network method for error correction. CGCUTS (Yang et al., 2016) combines multi-atlas labeling and random forest classification in the context of a graph cut framework to produce the segmentation. Van der Lijn et al. (van der Lijn et al., 2009) present a method that combines an appearance model and atlas registration. Carass et al. (Carass et al., 2018) summarize and compare several cerebellum sub-segmentation methods, highlighting CERES2 (Carass, Cuzzocreo, Han, Hernandez-Castillo, Rasser, Ganz, Beliveau, Dolz, Ben Ayed, Desrosiers, Thyreau, Romero, Coupé, Manjón, Fonov, Collins, Ying, Onyike, Crocetti, Landman, Mostofsky, Thompson, Prince, 2018, Romero, Coupé, Giraud, Ta, Fonov, Park, Chakravarty, Voineskos, Manjón, 2017) as the most performant ‘traditional’ (i.e. non deep-learning) approach. However, while image processing with CERES1 is supported online, CERES2 is unavailable to the scientific community.

The most recent cerebellum sub-segmentation tool, Anatomical Parcellation using a U-Net with Locally Constrained Optimization (ACAPULCO) (Han et al., 2020b), introduces a two-step deep learning method with two 3D convolutional neural networks (CNNs) to first localize and then sub-segment the cerebellum, outperforming the challenge winner CERES2 (Carass et al., 2018) in a head-to-head comparison. Preprocessing steps include bias field inhomogeneity correction and registration to MNI space. However, both the training and the evaluation procedure include some short-comings, e.g. by forcing nearest neighbor label interpolation both during training and evaluation, predominantly reducing detail in fine structures such as thin CWM strands. In fact, reported performance metrics were calculated entirely in MNI space, which required lossy nearest neighbor interpolation of manual reference labels to MNI space potentially mischaracterizing segmentation performance.

In contrast to ACAPULCO, our method does not require any preprocessing steps such as bias field correction or spatial (atlas) normalization/registration during inference. Moreover, to increase the anatomical variety in our training data, we employ various augmentation approaches, e.g. we generate realistic non-linear deformations via cross-subject registration of training images to various images from multiple datasets. To further improve the generalization of our model we pre-train the model on a compiled cross-study dataset. We examine the effect of data augmentations such as non-linear deformation and pre-training in several experiments. In our proposed method, the neural network architecture follows *FastSurferCNN*, a 2.5D approach in which three 2D networks for each axial, coronal, and sagittal view are trained and the final 3D prediction is created by view-aggregation (Henschel et al., 2020).

### 1.3 Contributions

This work presents five contributions:

• A detailed labeling protocol ensuring replicability and extensibility for the 25 cerebellar cortex labels and 2 cerebellar white matter segmentations including the fine branching, as well as a manual reference dataset of consensus cerebellar subsegmentation labels for training and testing.
• A training methodology with extensive data augmentation including realistic deformations to address the challenge of a small training dataset.
• Detailed method ablation to establish design choices in dedicated experiments on a subset of cases, not overlapping with the training or test sets.
• *CerebNet* consistently and significantly outperforms state-of-the-art cerebellum sub-segmentation methods with respect to accuracy and test-retest reliability.
• Sensitive *CerebNet* segmentations reproduce cerebellar atrophy effects in the pre-ataxic stage of spinocerebellar ataxia type 3 with a superior group separability.

## 2 Methods

We first describe the datasets for training, validation, and testing of *CerebNet*, then continue with the description of our method, and finally detail the evaluation.

### 2.1 Datasets

#### 2.1.1 CerebNet dataset

We assemble a diverse cerebellum sub-segmentation dataset for training, validation and testing of models based on acquisitions from ongoing observational studies. This superset includes participants equally distributed between healthy controls as well as pre-ataxic and ataxic SCA3 mutation carriers, thereby covering a broad range of different degrees of cerebellar atrophy.

*Participants* 32 T1-weighted MRI of SCA3 mutation carriers and healthy controls were acquired at 4 sites: Bonn and Aachen, Germany, Nijmegen, The Netherlands and Minneapolis, MN, US. All participants provided written informed consent according to the guidelines set by the local institutional review boards. Two cases with visible motion artifacts were excluded, resulting in the final CerebNet dataset of 20 SCA3 mutation carriers, covering the whole disease course of SCA3 from early pre-ataxic to late ataxic disease stages, and 10 healthy controls of the same age range. In Table 1, we report demographics (age, sex) and ataxia severity, assessed with the Scale for Assessment and Rating of Ataxia (SARA) (Schmitz-Hübsch et al., 2006) for the three groups. For SCA3 mutation carriers, we also report the CAG repeat length. To divide SCA3 mutation carriers into pre-ataxic (SARA <3, N=11) and ataxic individuals (SARA ≥3, N=9), we follow the established SARA cut-off value of 3, corresponding to the mean plus 2 standard deviations of the healthy control group distribution from the original SARA validation study (Schmitz-Hübsch et al., 2006).

**Table 1:** Demographic and characterizing data of the *CerebNet* data set cohort consisting of pre-ataxic and ataxic SCA3 mutation carriers as well as healthy controls (HC). ^1^Time to onset is given in years. The reported time from onset (defined as the first occurrence of gait disturbances) is given where available and for the remaining seven pre-ataxic mutation carriers, not yet experiencing gait disturbances, we estimated the time to onset following the model introduced by Tezenas et al. [42], which depends on both the number of CAG RL as well as the actual age; SD = standard deviation, CAG RL = CAG repeat length of the longer allele.

Group	N	**age** [years]	sex	SARA	**Time to ataxia onset^1^**	CAG RL
		mean ± SD [range]	m/f	mean ± SD [range]	mean ± SD [range]	mean ± SD
HC	10	43.9 ± 13.22 [22; 63]	4/6	0.3 ± 0.54 [0; 1.5]	n.a.	n.a.
pre-ataxic SCA3	11	31.6 ± 7.1 [20; 43]	4/7	1.4 ± 0.8 [0; 2.5]	-4.5 ± 6.4 [-13.8; 8.0]	72.4 ± 3.1
ataxic SCA3	9	44.6 ± 7.3 [32; 57]	6/3	12.6 ± 4.5 [7; 19]	8.4 ± 5.4 [1.0; 8.0]	70.89 ± 4.11

*MRI scans* All T1-weighted MRI were acquired as MPRAGE on 3T SIEMENS scanners (Siemens Medical Systems, Erlangen, Germany). All scans share an isotropic resolution of 1mm, FOV 256×256 and 192 slices, acquired in sagittal direction with a 32-channel head coil. Bonn (N=16, Skyra), Minnesota (N=7, Prisma Fit), and Aachen (N=4, Prisma) acquired at TR = 2500ms, TE = 4.37ms, TI = 1100ms, FA = 7°, while Nijmegen (N=4, Trio) acquired at TR = 2300ms, TE = 3.03ms, TI = 1100ms, FA = 8°.

*Segmentation Protocol* Following the Schmahmann atlas (Schmahmann et al., 1999) as anatomical reference, we define 27 disjoint macroscopic subsegments of the cerebellum. In addition to 20 hemispheric lobules (10 for each hemisphere), we include 5 vermis labels and two CWM labels (left and right). The cerebellar segmentation is divided into 6 hierarchical steps, gradually moving from large-scale structures to the subdivision of cerebellar lobules. First, we delineate the CGM cortex with an exact outer boundary separating CGM from cerebrospinal fluid and other subtentorial structures, such as cranial nerves (*Step 1*). In this step, any inwardly projecting CWM branches are ignored. Subsequently, the four lobes (*Step 2*), and the vermis (*Step 3*) are segmented. We conduct the sub-segmentation of the hemispheric lobules (*Step 4*) as well as the subdivision of the vermis (*Step 5*). Finally, the fine delineation of the CWM including its branches into the CGM cortex band and a consistent boundary towards the brainstem is drawn (*Step 6*). The detailed protocol is publicly available for reproducibility (Heinz et al., 2022).

**Fig. 1:**
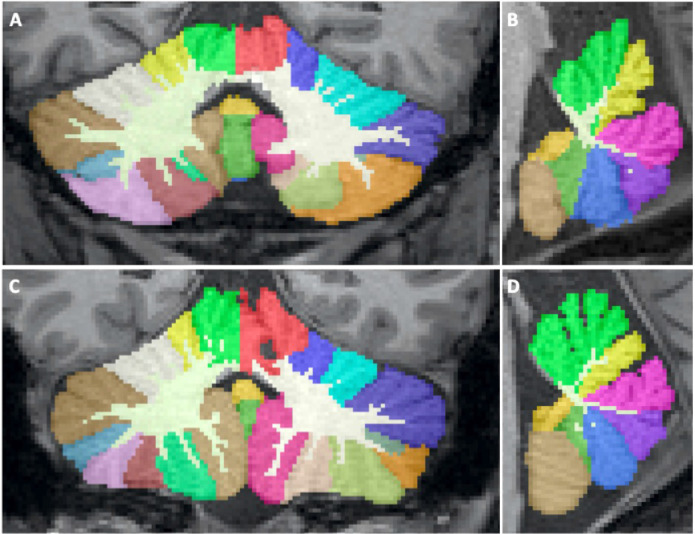
Segmentation examples of a fully automated segmentation of *CerebNet* in a healthy control (A, B) as well as a symptomatic SCA3 patient (C, D) projected onto a coronal and sagittal slice.

*Manual Reference Standard* The correct subdivision of the cerebellar cortex into lobules is critical, since the cerebellum shows a high morphological variability of its anatomical structure (Fig. 1). At the isotropic resolution of 1mm, it remains challenging to precisely determine whether a single small folia or branch belongs to one or an adjacent lobule. To address this, all lobular boundaries within the cerebellar cortex (*Step 1-5*) are subsegmented by two experienced raters on all MRIs independently. To unify differences between the two raters, cortex segmentations are reviewed by an interdisciplinary team consisting of the experienced raters as well as a neurologist and a neuroradiologist. A consensus was reached for all cases. Furthermore, a team of four trained raters delineated the fine-grained CGM/CWM boundary (*Step 6*). The final consensus segmentation together with the CWM delineations represents the manual reference standard for training, validation, and testing of our method. We split the participants contained in the final reference dataset into 18/4/8 for training, validation, and testing. For individual splits, we preserve the distribution of controls, pre-ataxic and ataxic participants.

#### 2.1.2 Cross-study pre-training dataset

For pre-training purposes, we compile a dataset of 160 T1-weighted images gathered from the Autism Brain Imaging Data Exchange II (ABIDE II) (Di Martino et al., 2017), the Alzheimers Disease Neuroimaging Initiative (ADNI) (Mueller et al., 2005), the UCLA Consortium for Neuropsychiatric Phenomics LA5c Study (LA5c) (Poldrack et al., 2016), the Open Access Series of Imaging Studies 1 and 2 (OASIS-1^3^ (Marcus et al., 2007) and OASIS-2 (Marcus et al., 2010)), and the Minimal Interval Resonance Imaging in Alzheimers Disease (MIRIAD) (Malone et al., 2013). For these 160 cases, we automatically generate cerebellar sub-segmentation labels using SUIT v3.3 (Diedrichsen, 2006, Diedrichsen, Balsters, Flavell, Cussans, Ramnani, 2009). SUIT is an atlas-based segmentation tool which provides segmentations of 28 sub-regions of the cerebellar cortex at the level of cerebellar lobules and a sub-segmentation of the vermis according to the Schmahmann atlas (Schmahmann et al., 1999). Since SUIT does not provide segmentations for CWM, we additionally process all 160 images with *FreeSurfer* (FS) (Fischl et al., 2002) and merge FS-generated CWM with the cerebellar sub-regions labels from SUIT. Gaps between cerebellar CWM and CGM are resolved by mapping them to the nearest CGM structure. The compiled external dataset is split into 140 training and 20 validation cases and is exclusively used for pre-training of our model.

#### 2.1.3 Deformation dataset for augmentation

To increase variability of our training data, we generate realistic non-linear deformations for data augmentation. For this we generated an auxiliary dataset of 100 cases selected from ABIDE II (Di Martino et al., 2017), ADNI (Mueller et al., 2005), LA5c (Poldrack et al., 2016), OASIS-1^3^ (Marcus et al., 2007) and OASIS-2 (Marcus et al., 2010), MIRIAD (Malone et al., 2013), and the Human Connectome Project (HCP) (Van Essen et al., 2012).

#### 2.1.4 Test-retest dataset

We use the OASIS-1 (reliability subset) (Marcus et al., 2007) and Kirby (Landman et al., 2011) datasets for test-retest analysis. OASIS-1 contains 20 participants that were scanned no more than 90 days apart (all except 5 less than 30 days). The Kirby dataset consists of scan-rescan MPRAGE images of 21 healthy participants with one hour break between scanning sessions.

### 2.2 Cerebellar sub-segmentation method

This section introduces our cerebellar sub-segmentation pipeline, consisting of an initial localization step to extract the relevant cerebellum region, a subsequent multi-view ensemble for CNN-based segmentation, and a final view-aggregation step to merge the predictions. The pipeline accepts unprocessed 1.0mm T1-weighted images and outputs segmentation maps and tabulated volume reports. To achieve high accuracy, the relatively small size of the manual reference standard requires special consideration. We address it by pre-training with a representative cross-study dataset as well as applying data augmentation. Specifically, intensity and spatial data augmentation techniques such as realistic deformations increase the diversity presented to the network during training and, thus, its performance.

*Localization* To constrain the sub-segmentation network to the cerebellum and reduce memory and computational requirements, we crop a bounding box of 128×128×128 isotropic 1mm voxels containing both cerebelli. The bounding box is placed symmetrically around the full cerebellar region obtained from a quick single-view (coronal) *FastSurfer* segmentation (Henschel et al., 2020). A visual inspection of this localization approach confirms the cerebellum is always correctly localized and fully contained within the bounding box in all cases.

*Cerebellar Sub-segmentation Network* The method for cerebellum sub-segmentation follows *FastSurfer* (Henschel et al., 2020). Briefly, in its 2.5D approach, *FastSurfer* utilizes an ensemble of three two-dimensional CNNs (*FastSurferCNN*) – each of these processing the MRI images sliced in a different direction (axial, coronal, and sagittal views). A final view-aggregation step combines the resulting label probability maps in probability space. *FastSurferCNN* is a U-Net-based fully CNN architecture with a dense encoder and decoder block per depth-level. In contrast to its predecessor (Guha Roy et al., 2019), the architecture extends the dense blocks and unpooling operations with a local competition approach (Estrada, Conjeti, Ahmad, Navab, Reuter, 2018, Estrada, Lu, Conjeti, Orozco-Ruiz, Panos-Willuhn, Breteler, Reuter, 2020) and gathers information in the third dimension via spatial information aggregation (SPI). The SPI approach provides the network with a wider volumetric context by stacking additional three preceding and three succeeding neighboring slices for a total of 7 input channels. Both the view aggregation and the SPI approach together allow the method to process 3D information, while at the same time retaining the computational advantages of 2D networks, primarily lower memory requirements and sample efficiency, i.e. 1. a lower number of parameters compared to 3D networks and 2. 3D MRI are split into slices increasing the number of samples presented to the network.

*Spatial Augmentations* Spatial augmentations such as flipping, translation, rotation, and scaling were used during training to improve the robustness of our model. We encode these transformation as a 3×3 in-slice transformation matrix in homogeneous coordinates with coefficients uniformly sampled from predefined ranges. Random offsets along the in-slice-axes for translation are selected from -12 to 12mm to simulate cerebellum centroid variation. We sample the scaling factor from 0.95 to 1.2. The image is rotated in-slice with respect to its center with angles uniformly sampled from -20° to 20°. We also apply a random left-right flip, i.e., both the image and its labels are mirrored, but label IDs are swapped with respect to the mid-plane separating the two hemispheres keeping left-labels on the left.

*Augmentation with non-linear Deformation* To increase variability of our training data, we perform static augmentation with 500 non-linearly deformed training images. For this, we first non-linearly register each image of the Deformation (see Section 2.1.3) to 5 randomly selected images from the training split of the CerebNet dataset (see Section 2.1.1) using ANTs v2.3.1 (Avants et al., 2008). We ensure each manually labeled *CerebNet* case is at least paired once. For each of the resulting 500 anatomically realistic deformation fields, we then map both image and manual label from the training split of the CerebNet dataset using the obtained deformation field. In effect, this procedure drastically increases the anatomical variance presented to the network during training.

*Intensity Augmentations* Random MRI magnetic field inhomogeneities are synthesized and linearly superimposed to the images to increase the robustness of the model to bias field artifacts. We generate the augmented inhomogeneity field by linear-combination of randomly weighted cubic polynomial basis functions (Van Leemput et al., 1999). The coefficients of the basis functions are uniformly sampled from a -0.5 to 0.5 range.

### 2.3 Metrics for evaluation

To establish the quality and accuracy of *CerebNet* with respect to volumetric and geometric features, we evaluate the resulting segmentations with three common segmentation metrics: The *Dice Score*, calculated as the general label overlap, is well established as a good compromise between volumetric and geometric segmentation properties; the *Hausdorff Distance* serves as a metric for geometric and spatial similarity, and finally the *Volume Similarity* completely ignores overlap and spatial distance, but most directly evaluates the reliability for volumetric measures commonly used in statistical modeling.

*Dice Score* The Dice score (Dice) (Dice, 1945, Sørensen, Julius, 1948), is one of the most frequently used metrics in validating semantic segmentations. If P and G are the segmentation maps of the network prediction and ground-truth respectively, then Dice is defined as:

(1) $$\text{Dice}=2\times \frac{\left|G\cap P\right|}{\left|G|+|P\right|}\text{,}$$

where |.| represents cardinality. It measures overlap of 3D volumes on a scale between 0 and 1, where a value of 1 indicates exact agreement and 0 disjoint segmentations.

*Hausdorff Distance* To evaluate the quality of segmentation boundaries we calculate the distance between the manual and the automatic segmentation boundaries. In particular, this distance metric allows to test the overall accuracy of the boundary delineation emphasizing the correct contour. As this distance-metric decreases, segmentation boundaries more closely correspond to each other locally, i.e. more agreement of geometric details. Boundary distances can be quantified by the standard Hausdorff Distance (HD) or the Robust Hausdorff Distance (HD95). The standard HD measures the maximum distance and therefore is strongly affected by local outliers. HD95 – the 95% percentile of distances between surfaces (Huttenlocher et al., 1993) – is less sensitive to outliers and consequently more informative when analyzing the general trend. Formally, for boundaries $B_{G}$ (of the ground-truth label map G) and $B_{P}$ (of its predicted correspondent), we use their distances $D_{G↔P}=\left\{\min_{g\in B_{G}}\;d\left(p,g\right)\;|\;\forall p\in B_{P}\right\}\cap \left\{\min_{p\in B_{P}}\;d\left(p,g\right)\;|\;\forall g\in B_{G}\right\}$ to compute HD and HD95 as $d_{\text{HD}}=\max D_{G↔P}$ and $P(d<d_{\text{HD95}})<0.95$, $d\in D_{G↔P}$.

*Volume Similarity* Volume similarity ($vol_{\text{sim}}$) compares the absolute volume difference with the sum of volumes. Given $V_{G}$ and $V_{P}$, the volumes of the ground-truth and predicted segmentations (G and P), $vol_{\text{sim}}$ is calculated as

(2) $$vol_{\text{sim}}=1-\frac{\left|V_{G}-V_{P}\right|}{V_{G}+V_{P}}\;\text{.}$$

Since this metric ignores overlap and geometric information, the optimal similarity (a value of 1) can be achieved for two segmentations of the same size, even if their spatial overlap is zero. However, its independence from spatial correspondence enables cross-acquisition comparison, e.g. for test-retest analysis, without requiring image alignment.

*Intraclass Correlation Coefficient* The Intraclass Correlation Coefficient (ICC) (Shrout and Fleiss, 1979) evaluates the reliability and agreement between measurements. Its values range from 0 to 1 with larger values representing higher reliability. We also compute the 95% confidence interval around the ICC. For test-retest scenarios, we calculate the ICC as a measure of agreement between two repeated scans of the same participant (relative agreement, single fixed rater). Since scans are acquired in close temporal proximity, we assume only little volumetric changes and thus a high ICC.

### 2.4 Implementation details

Here, we detail the training of *CerebNet* and our adaptations of state-of-the-art methods to establish compatibility with our labeling protocol.

*CerebNet Training* We train each network for axial, coronal, sagittal views independently for 70 epochs with a batch size of 128 using one NVIDIA Tesla V100 GPU with 32 GB RAM. We use the AdamW (Kingma, Ba, 2015, Loshchilov, Hutter, 2019) optimizer with a weight decay of $10^{-4}$ and an initial learning rate (LR) of 0.01. The *reduce on plateau* strategy for scheduling updates the LR based on the Dice score on the validation set. This strategy reduces the LR by a factor of 0.01, if there is no improvement in Dice score for 4 epochs.

*ACAPULCO Re-Training* In order to detach protocol and training data differences from method features, we retrain the deep learning method ACAPULCO (Han et al., 2020b) with our data following identical splits, hereafter referred to as ACAPULCO^rt^. For this, we start with the published method code of ACAPULCO and configure the dataset loader to accept our dataset. Since we do not have access to the ACAPULCO training data, training our method on their data for comparison is not possible. We trained ACAPULCO with the publicly available source code and therein defined hyperparameters.

*SUIT+FS* The leading traditional method (Carass et al., 2018), SUIT (Diedrichsen et al., 2009) does not rely on a deep learning approach and is only compatible with the CerebNet labels after combination with *FreeSurfer* (Fischl et al., 2002). In analogy to Section 2.1.2, we therefore merge SUIT cerebellum sub-segmentation labels with *FreeSurfer*’s CWM segmentation (SUIT+FS) to obtain the full set of labels.

## 3 Results

We report detailed results for multiple experiments to assess the performance of *CerebNet*. First, we ablatively establish the configuration and parameters of *CerebNet* on a validation hold-out set. Second, keeping method parameters fixed from here on, we compare the average performance of *CerebNet* with the state-of-the-art using four volumetric and geometric metrics: the Dice Score, two Hausdorff distances, and volume similarity. We investigate regional performance differences of these methods for all cerebellar sub-structures. Third, we contextualize the accuracy of *CerebNet* with differences between raters. Fourth, we compare the test-retest reliability of *CerebNet* with the state-of-the-art method ACAPULCO (Han et al., 2020b). Finally, we validate whether *CerebNet* reproduces known group differences between pre-ataxic and ataxic patients and healthy controls.

### 3.1 Ablation experiments

**Fig. 2:**
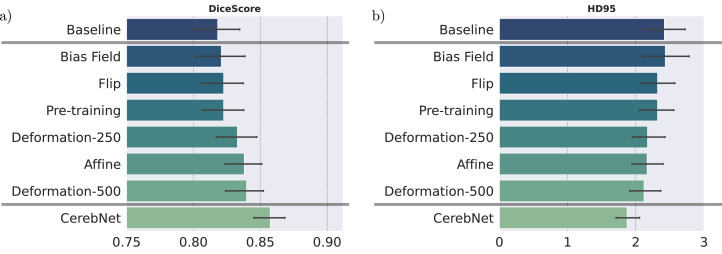
Dice score (larger values are better) and Robust Hausdorff Distances (HD95, smaller values are better) on validation cases for comparison of baseline, individual method contributions (not cumulative) and *CerebNet. CerebNet* combines multiple data augmentations with pre-training on a representative cross-study dataset. Deformation-250/500 indicates the number of realistic deformation fields used for static augmentation. The baseline model is our network without augmentation or pre-training. Error bars indicate 95% confidence intervals.

We perform several experiments to determine, how different changes to the data augmentation impact the performance of our method. In specific, we isolate the individual effects of different data augmentation methods and pre-training. We assess random flipping (Flip), bias field, affine deformation, and realistic non-linear deformation (Deformation-N, we test N=250 and N=500 deformation fields). While the baseline foregoes all data augmentation and pre-training, *CerebNet* combines all data augmentations with pre-training. All individual contributions improve results over the baseline (Fig. 2) in both Dice and Robust Hausdorff Distance (HD95) evaluations. Finally, the combination of all contributions clearly improves the results over any individual approach. We exclusively evaluate on validation cases for this analysis to avoid data-leakage.

### 3.2 Comparison with the state-of-the-art

**Fig. 3:**
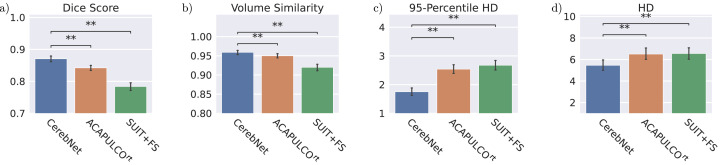
Comparison of mean a) Dice score (larger values are better), b) Volume similarity (larger values are better), c) Robust Hausdorff Distance (HD95, smaller values are better), and d) Hausdorff Distance (HD, smaller values are better) over all structures and participants. *CerebNet* outperforms both ACAPULCO^rt^ (which is retrained on our dataset for direct comparison) and SUIT+FS. Error bars indicate 95% confidence intervals. Statistical significance for all results is confirmed by two-sided non-parametric Wilcoxon signed-rank tests (**: p<.01).

In a summary evaluation, we compare the overall performance of *CerebNet*, ACAPULCO^rt^ (Han et al., 2020b) and SUIT+FS (Diedrichsen, 2006, Fischl, Salat, Busa, Albert, Dieterich, Haselgrove, van der Kouwe, Killiany, Kennedy, Klaveness, Montillo, Makris, Rosen, Dale, 2002) on the test subset of the CerebNet dataset (Section 2.1.1). On average across all segmented structures, *CerebNet* achieves a 0.870 per-structure Dice score and a 1.742mm Robust Hausdorff distance, which is the 95% percentile of surface-to-surface distances. In comparison with both state-of-the-art approaches, *CerebNet* outperforms either approach significantly in all four metrics (p<.01, see Fig. 3): the Dice score, Hausdorff distance (HD), Robust Hausdorff Distance (HD95) and volume similarity.

Results for individual structures are very consistent across all four metrics. Therefore, we focus further analysis and discussion on the Dice score and the Robust Hausdorff Distance. Specifically, we favor the robust implementation, since its robustness to outliers better reflects the accuracy across the surface, yet the high margin of 95% ensures larger structures (like CWM strands) are captured. Additionally, conclusions and reported significance values (derived by a Wilcoxon signed-ranked test) are completely independent of the choice of Hausdorff metric.

**Fig. 4:**
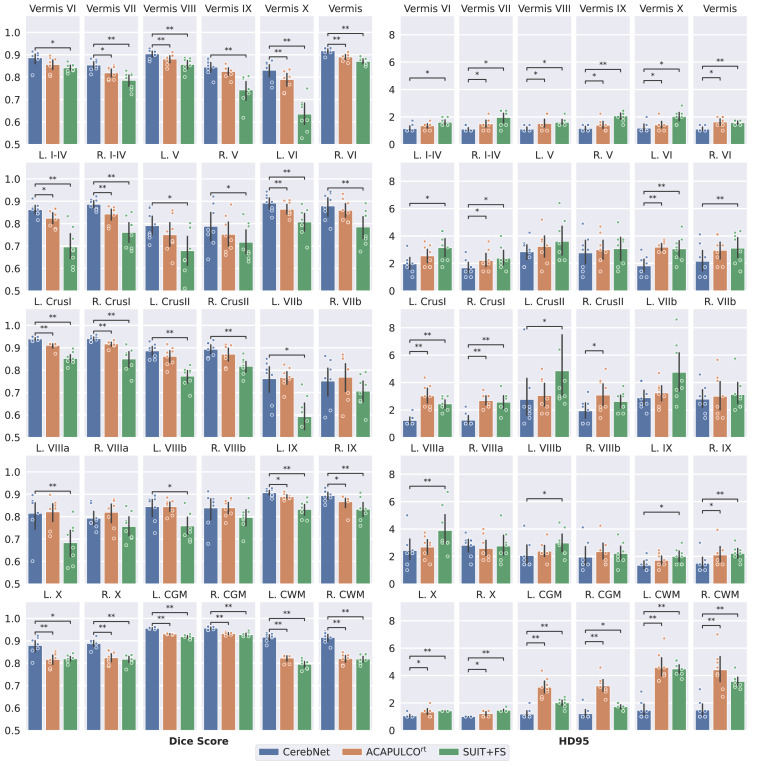
Dice score (larger values are better) and Robust Hausdorff Distance (HD95) (smaller values are better) per sub-structure for *CerebNet*, ACAPULCO^rt^ and SUIT+FS. Illustrations show the cross-subject average of the metric (bar) and corresponding, bootstrapped 95% confidence intervals (error bars), data points (eight per bar, may overlap) as well as the significance level calculated by a Wilcoxon signed-rank test (*:p<.05 and **:p<.01). CGM: Cerebellar Gray Matter; CWM: Cerebellar White Matter.

*Dice Score CerebNet* surpasses a 0.75 Dice score for all 27 individual structures and exceeds 0.95 Dice for the joint CWM. In fact, the least performing structures (specifically lobes VIIb and VIIIa/b) are “thin structures” sharing predominantly hard to define boundaries with other gray matter regions. *CerebNet* outperforms ACAPULCO^rt^ in 22 of 27 individual structures. In 14 out of 27 structures the improvement is significant (10 times p<.01 and 4 times p<.05, Fig. 4). For regions, with better performance of ACAPULCO^rt^, the difference is usually small and never significant. *CerebNet* also significantly improves over ACAPULCO^rt^ segmentations for both merged gray matter and merged vermis regions (p<.01, Fig. 4). In comparison to the traditional SUIT+FS method, *CerebNet* always achieves better Dice scores, which are also statistically significant for all but three sub-structures (Fig. 4).

*Robust Hausdorff Distance (HD95)* On average, *CerebNet* achieves a HD95 distance of 1.742mm improving substantially over ACAPULCO^rt^ by 0.779mm. Across different vermis regions, the Crus I and lobe X regions and the merged gray matter region, *CerebNet* even exceeds a 1.25mm threshold. Larger distances remain in the lobes, where hard to reproduce lobe-to-lobe boundaries dominate the evaluation. Across all 27 structures, *CerebNet* outperforms ACAPULCO^rt^ in 26 of 27 structures (significantly for 14 structures, 5 times p<.01, 9 times p<.05, Fig. 4). In fact, the large performance differences for the merged CWM and CGM (both ⪆2mm) clearly indicate the differences between methods (Fig. 4). Compared with SUIT+FS, *CerebNet* demonstrates a superior performance consistently.

To quantify the robustness of *CerebNet*, we perform an outlier analysis for the Dice and Robust Hausdorff results. All data points are within 2.5 standard deviations of the per class mean.

### 3.3 Inter-rater reproducibility

**Fig. 5:**
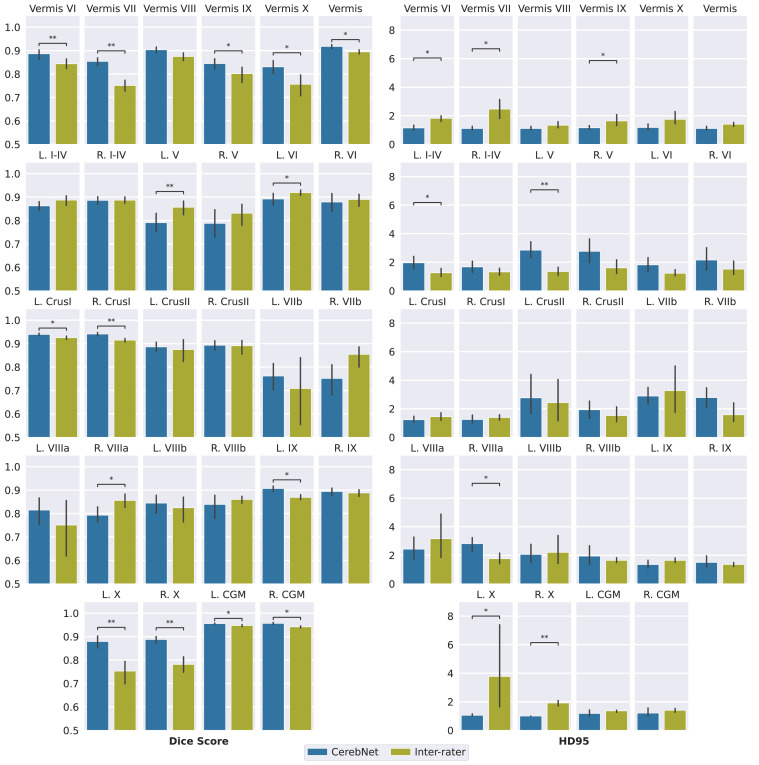
Comparison of Inter-rater reliability and *CerebNet* by Dice score and Robust Hausdorff Distance (HD95) per sub-structure. Error bars indicate 95% confidence intervals. CGM is Cerebellar Gray Matter and CWM is Cerebellar White Matter (*:p<.05 and **:p<.01).

In our experience, delineation of cerebellar sub-structures, manual or automatic, is a challenging task due to the inherent uncertainty and lack of information to determine boundaries between cerebellar lobules even at 1mm isotropic resolution. To evaluate the *CerebNet* performance in the context of the reliability of manual segmentation, we analyze *CerebNet* segmentation errors together with the inter-rater variability. Figs. 4 and 5 share the same evaluation results for *CerebNet* in both cases comparing *CerebNet* predictions with the final “consensus segmentation” (after Step 6, see Section 2.1.1). However, for best annotation quality, labels from multiple raters are merged and harmonized in Step 6 of the protocol (see Section 2.1.1). To consistently and comparably represent the inter-rater reliability, we compare labels from one rater prior to this harmonization (after Step 5) to the “consensus”. Since Step 5 data also does not include CWM labels, we exclude segmentation errors along the CGM/CWM boundary in the inter-rater evaluation (i.e. we mask out the CWM as defined in the “consensus”) and only consider CGM regions for evaluation.

To evaluate the inter-rater variability, we utilize the Dice score and the Robust Hausdorff Distance (HD95). Fig. 5 illustrates per-region *CerebNet* and inter-rater Dice scores and HD95 on the *CerebNet* test set. Both volumetric and geometric segmentation scores are strongly correlated and – in most cases – at similar levels. Specifically, lower *CerebNet* performance values also map to lower inter-rater reliability in lobes V, VIIb and VIIIa/b. Results to lobe X as well as vermis VII/IX/X are outliers to this observation, where *CerebNet* provides good segmentations despite – in comparison – low inter-rater reliability.

### 3.4 Test-retest reliability

With the substantial time and labor requirements of manual segmentation, crucial external validation of methods is not easily possible. However, test-retest datasets with multiple scans of the same underlying anatomy and acquisition/machine properties offer the opportunity to test the reliability of methods. The OASIS-1 reliability dataset (Marcus et al., 2007) and Kirby dataset (Landman et al., 2011) are not only acquired at sites and in studies independent of the CerebNet dataset, but also feature 1.5T Siemens and 3T Philips scanners, respectively. To avoid influences of potentially error-prone image registration and interpolation, only per-structure volumes will be compared, as all geometric analysis would require alignment of baseline and follow-up scans.

**Fig. 6:**
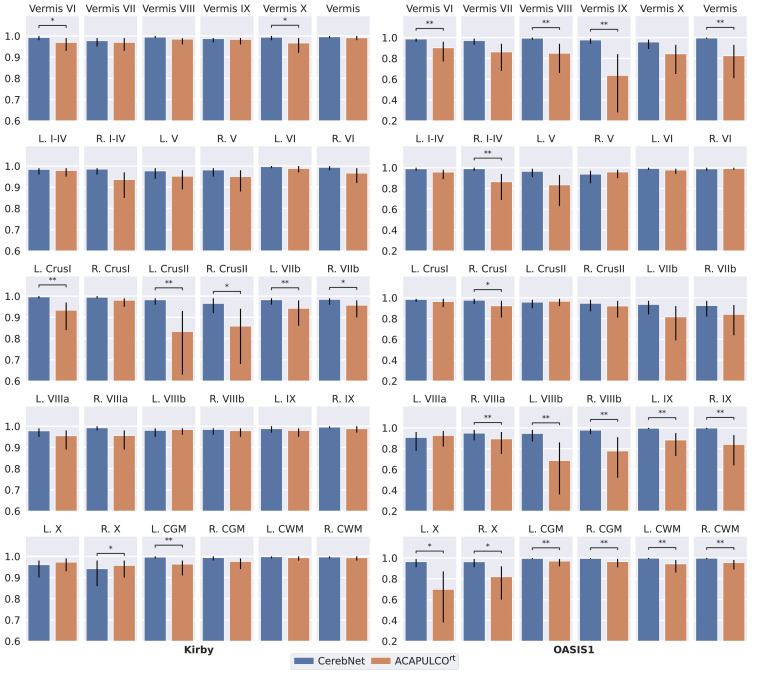
Intraclass correlation coefficient (ICC) on volume of Kirby and OASIS1 datasets for test-retest analysis. Error bars indicate the 95% confidence interval. Statistical significance is calculated with a two-sided non-parametric Wilcoxon signed-rank test over the absolute volume difference, since ICC values cannot provide significance information. * and ** annotations represent statistical significance for better volume consistency with p<.05 and p<.01, respectively.

Here, we compare the reliability of regional volumes with the intra-class correlation coefficient (ICC) and the volume differences derived from the two test-retest images. In Fig. 6, we plot the ICC values (and its 95% confidence interval) of *CerebNet* and ACAPULCO^rt^ for the two datasets. Statistical significance tests, however, are directly performed on volume differences using a Wilcoxon signed-rank test to compare the methods. The ICCs of *CerebNet* and ACAPULCO^rt^ range between 0.635 and 0.997 across both datasets with – in most cases – more consistent results (higher ICC) for *CerebNet*. In fact, the ICC is superior for *CerebNet* over ACAPULCO^rt^ in 24 of 27 sub-structures for the Kirby data and in 23 out of 27 sub-structures for the OASIS1 data set set as well as for the combined regions of the vermis and the left and right hemispheric CGM. This difference was significant in 17 (9) out of all 30 structures for the OASIS1 (Kirby) data set (only once in favor of ACAPULCO^rt^,  Fig. 6). In particular, *CerebNet* was more consistent as evidenced by much lower standard deviations and smaller 95% confidence intervals of the ICC for each sub-structure in comparison to ACAPULCO^rt^ (Fig. 6).

### 3.5 Volumetric changes in pre-ataxic and ataxic spinocerebellar ataxia type 3 (SCA3)

We analyzed the cerebellar volumes of 109 SCA3 mutation carriers and 41 healthy controls (HC), who are participants of ongoing observational studies and gave their written informed consent. MRI were acquired at 7 EU and 2 US sites. All T1-weighted images were acquired on 3T SIEMENS scanners (Siemens Medical Systems, Erlangen, Germany) with an isotropic resolution of 1mm. To establish generalizability of *CerebNet* to this dataset, we visually inspect a random subset of 5 cases per group (total N=15) finding good segmentation quality with no outliers.

To investigate group differences between pre-ataxic and ataxic SCA3 as well as healthy controls, we used a linear mixed-effects model with the co-variables age and estimated total intracranial volume (eTIV) as well as group (pre-ataxic SCA3, ataxic SCA3 and HC) and sex as fixed and scanner as random factors, respectively. Ataxia severity was assessed with the Scale for Assessment and Rating of Ataxia (SARA) (Schmitz-Hübsch et al., 2006). We applied the common SARA cut-off value of 3 to divide the group of SCA3 mutation carriers into pre-ataxic (SARA <3) and ataxic (SARA ≥3) individuals (Jacobi et al., 2020). The eTIV was assessed using *FreeSurfer* 6.0 (Buckner et al., 2004). Cerebellar volumes were compared between pre-ataxic SCA3 (N=42, mean age 38.02 years, 62.91% female, mean SARA 1.25) and ataxic SCA3 (N=67, mean age 49.94 years, 35.82% female, mean SARA 12.05) as well as healthy controls (N=41, mean age 43.95, female 43.90%, mean SARA 0.27). In the post-hoc analyses of pairwise comparisons, we applied Bonferroni correction for multiple comparisons. P-values smaller than p<.05 after Bonferroni correction were considered significant.

For *CerebNet*-derived per-region volumes, pre-ataxic SCA3 mutation carriers already showed significant volume reduction in comparison to HC in the right lobules I-IV, left and right lobule X, vermis IX as well as the left and right CWM. We detected significant volume reduction of ataxic patients in comparison to pre-ataxic SCA3 mutation carriers in left and right lobule VI, Crus II, VIIb, VIIIa and left VIIIb, left and right X and the left and right CWM. These results reaffirm that cerebellar neurodegeneration already starts before the clinical onset of the disease and is ongoing throughout the disease course with a very early and continuous involvement of cerebellar white matter.

**Fig. 7:**
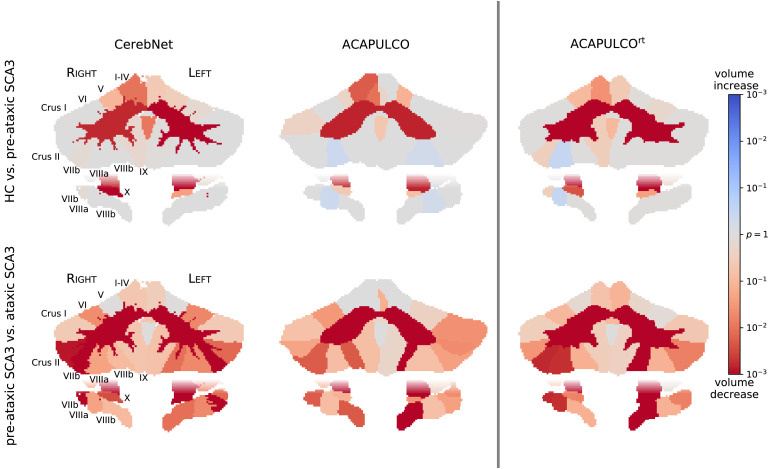
Map of volume change in HC vs. pre-ataxic SCA3 (top) and pre-ataxic vs. ataxic SCA3 (bottom). Per-region p-values of the respective group comparisons are shown for 3 different methods: *CerebNet*, ACAPULCO (as distributed by Han et al., 2020b) and ACAPULCO^rt^ (ACAPULCO retrained on our dataset). Red colors indicate atrophy, blue colors indicate volume increase (color saturation corresponds to significance). (For interpretation of the references to colour in this figure legend, the reader is referred to the web version of this article.)

In Fig. 7, we evaluate the power of our neuro-morphometric measures to separate between different groups: HC and pre-ataxic SCA3 mutation carriers (top) as well as pre-ataxic SCA3 and ataxic SCA3 (bottom). While in a direct competition of methods, only *CerebNet* and the original ACAPULCO (Han et al., 2020b) are available publicly, we also include ACAPULCO^rt^ (which is retrained with our labels, see Section 2.4) to illustrate the impact of both our high-quality training data and its interaction with our segmentation pipeline. A clear difference between the methods is already apparent in the varying details of the highly significant CWM segmentations and its branches. Even ACAPULCO^rt^ does not achieve the degree of detail available in *CerebNet*, in spite of it using the same training data. While a direct comparison of p-values is usually not possible, here it is meaningful as the methods operate on exactly the same input images. This is because the p-values of the group effect are monotonically connected to the absolute value of the t-statistic (effect size divided by standard error). More significant effects, i.e. smaller p-values illustrated in Fig. 7 by more saturated colors, indicate better group separation. In the group comparison of pre-ataxic mutation carriers to HC, ACAPULCO showed unexpected, non-significant volume increases in two structures and ACAPULCO^rt^ in one structure (blue regions in Fig. 7). Comparing the p-values for group separation between pre-ataxic SCA3 mutation carriers and healthy controls, *CerebNet* showed smaller p-values in more structures than ACAPULCO (10 versus 4) as well as ACAPULCO^rt^ (9 versus 6). For the group separation between pre-ataxic and ataxic SCA3 mutation carriers, *CerebNet* showed smaller p-values in 15 structures compared to 6 for ACAPULCO and 13 compared to 11 for ACAPULCO^rt^. Given that the true group differences for each sub-structure are unknown, these results cannot establish a final superiority, but they can assure that known and expected effects can be reliably detected and that this signal is recovered most strongly with *CerebNet*.

## 4 Discussion

Neuroanatomical volumetry is a promising imaging biomarker candidate to assess progressive neurodegeneration in clinical trials. The advantages are, first, that non-invasive T1-weighted MRI is widely available, second, that precise quantitative estimates can aid studies into disease progression even at early stages, and third, that these volume estimates permit assessing subtle changes to quantify atrophy rates in various disease stages and effects of potential interventions and disease modifying therapies. Especially quantitative estimates of cerebellar structures are highly relevant for studying ataxia, in particular for those ataxia disorders where clinical trials have already been initiated, such as SCA3. Therefore, with this work, we introduce a multi-stage protocol for reliable and repeatable cerebellum segmentation with carefully drawn and quality-assured boundaries, establish a manually segmented reference dataset, and develop and validate *CerebNet*, a fast and accurate method to automatically sub-segment the cerebellum into its lobules and the cerebellar WM from a T1-weighted MRI.

Our method *CerebNet* employs a *FastSurferCNN* deep-learning model customized to our cerebellum training dataset. In contrast to state-of-the-art methods (Carass, Cuzzocreo, Han, Hernandez-Castillo, Rasser, Ganz, Beliveau, Dolz, Ben Ayed, Desrosiers, Thyreau, Romero, Coupé, Manjón, Fonov, Collins, Ying, Onyike, Crocetti, Landman, Mostofsky, Thompson, Prince, 2018, Diedrichsen, 2006, Han, Carass, He, Prince, 2020), *CerebNet* does not require any preprocessing, such as spatial normalization or bias field correction, thus preserving sufficient detail to segment even the fine branches of the white matter and simultaneously allowing rapid processing at only 12 seconds per MRI with one GPU (Nvidia Titan Xp). Fast MRI segmentation in general opens up multiple avenues of potential applications, ranging from direct feedback or field-of-view localization during image acquisition or fast clinical decision support by quantitative personalized measurements. In addition to speed, we demonstrate in an extensive validation that the *CerebNet* pipeline outperforms state-of-the-art approaches and provides detailed segmentation masks especially for white matter strands.

Our quantitative analysis illustrates *CerebNet*’s superior segmentation quality in both volumetric and geometric metrics. Furthermore, we demonstrate *CerebNet*’s superior test-retest reliability and show-case its utility to down-stream group analysis: While clinical scales lack sensitivity in pre-ataxic cases, simply due to the absence of symptoms, *CerebNet* reliably identifies patterns of cerebellar degeneration consistent with previous studies (Faber, Giordano, Jiang, Kindler, Spottke, Acosta-Cabronero, Nestor, Machts, Düzel, Vielhaber, Speck, Dudesek, Kamm, Scheef, Klockgether, 2020, Kim, Kim, Lee, Lee, 2021, Rezende, de Paiva, Martinez, Lopes-Cendes, Pedroso, Barsottini, Cendes, França, 2018). Consequently volumetric estimates of the cerebellum, especially subtle longitudinal changes, are promising imaging biomarker candidates to assess the effect of preventive genetic therapies during the pre-ataxic stage and might play a central role as stratification markers or even as secondary outcome parameters in clinical trials.

A qualitative inspection of the predicted segmentation maps illustrates the different character of the presented pipelines. *CerebNet*-derived segmentation maps feature the highest level of detail, especially visible at the intricate boundary between CWM and CGM (see Fig. 7). In fact, comparing predictions of *CerebNet*, ACAPULCO and ACAPULCO^rt^^4^, we find that the level of detail of ACAPULCO^rt^ lies between *CerebNet* and ACAPULCO (see Fig. 7), highlighting both the added value of our dataset with its manual segmentations (ACAPULCO^rt^ vs. ACAPULCO) and of our method (*CerebNet* vs. ACAPULCO^rt^). In contrast to volumetric analyses, which are relatively robust to limited detail in segmentation maps, structural and geometric analyses, including thickness analysis, rely on accurate and detailed boundaries (Sörös et al., 2021). Because of the fine-grained furcations of CWM, the delineation of the CGM/CWM boundary is particularly challenging both for manual raters and automated methods. *CerebNet* especially improves these boundaries as proven by significantly improved Dice and Robust Hausdorff metrics over both ACAPULCO^rt^ and FreeSurfer (see L./R. CGM/CWM in Fig. 4).

In general, a critical limitation of learning-based approaches remains in the uncertain generalizability beyond images similar to those encountered during training. This limitation also applies to *CerebNet*. While our reference dataset features diversity in terms of severity of ataxia and cerebellar atrophy, the generalizability to other datasets is not guaranteed, since we only included T1w MRI of healthy controls and SCA3 mutation carriers acquired on SIEMENS scanners. Therefore, as for any method, dedicated experimental validation is required to confirm the validity under differing conditions, i.e. at least rigorous, manual quality checks of generated segmentations. Given the convincing test-retest performance (c.f. Fig. 6 Kirby dataset, which was acquired with Philips scanners), we are optimistic *CerebNet*’s extensive augmentation may already enable basic generalizability to other scan-settings. Furthermore, we visually inspected automatically generated segmentations of several clinically diagnosed sporadic and hereditary ataxias to verify whether *CerebNet* generalizes to other pathologies (N=14: two randomly selected cases of MSA-C, RFC1, SCA1, SCA2 and SCA6, AOA2 as well as one case of SYNE1 and CTX each, including cases with severe atrophy, see also Fig. 9 in the Appendix). While not a formal validation for these pathologies, we found the segmentation quality among these cases comparable to our SCA3 cases without fails or unacceptable quality, further supporting the generalizability of *CerebNet*.

Obviously, volumetric analyses of other sporadic and hereditary neurodegenerative ataxias are canonical further research questions. Moreover, *CerebNet* may enable cerebellar analyses of aging, non-motor diseases (e.g. Alzheimer’s disease or attention deficit hyperactivity disorder) and combined analyses of imaging and neuropsychological data. For these applications the focus may shift to parts of the cerebellum primarily involved in the adaptive control of non-motor processes. Since the functional representation of cognitive tasks is oriented across lobules along a parasagittal axis (King et al., 2019), the utility of segmentation along the anatomical boundaries of the hemispheric lobules is unclear for studies of the cerebellar involvement in cognitive and emotional processes.

In summary, *CerebNet* offers significant improvements and advantages for users in terms of runtime, accuracy, reliability and sensitivity to subtle cerebellar atrophy. Thus, we are confident, that *CerebNet* will enable and simplify the detailed morphometric analysis of the cerebellum.

## Acknowledgments

We would like to thank Beate Brol, Tim Elter, Isabelle Finkel, and Sophia Wismeth for their contribution to the manual segmentation. This work was supported by the National Ataxia Foundations SCA Young Investigator Award as well as by DZNE institutional funds, by the Federal Ministry of Education and Research of Germany (031L0206, 01GQ1801), and by NIH (R01 LM012719, R01 AG064027, R56 MH121426, and P41 EB030006). JF is fellow of the Hertie Network of excellence in clinical Neuroscience. This publication is an outcome of ESMI, an EU Joint Programme - Neurodegenerative Disease Research (JPND) project (see www.jpnd.eu). The project is supported through the following funding organisations under the aegis of JPND: Germany, Federal Ministry of Education and Research (BMBF; funding codes 01ED1602A/B); Netherlands, The Netherlands Organisation for Health Research and Development; Portugal, Foundation for Science and Technology and Regional Fund for Science and Technology of the Azores; United Kingdom, Medical Research Council. This project has received funding from the European Unions Horizon 2020 research and innovation programme under grant agreement No 643417. For the contribution of the Minnesota site, this work was in part supported by the National Ataxia Foundation and the National Institute of Neurological Disorders and Stroke (NINDS) grant R01NS080816. The Center for Magnetic Resonance Research is supported by the National Institute of Biomedical Imaging and Bioengineering (NIBIB) grant P41 EB027061, and the Institutional Center Cores for Advanced Neuroimaging award P30 NS076408 and S10OD017974 grant. Data used in the preparation of this article for pre-training and augmentation were obtained in part by the OASIS Cross-Sectional with principal investigators D. Marcus, R, Buckner, J, Csernansky J. Morris; P50 AG05681, P01 AG03991, P01 AG026276, R01 AG021910, P20 MH071616, U24 RR021382, and OASIS: Longitudinal: Principal Investigators: D. Marcus, R, Buckner, J. Csernansky, J. Morris; P50 AG05681, P01 AG03991, P01 AG026276, R01 AG021910, P20 MH071616, U24 RR021382. Further, data used in the preparation of this article were obtained from the MIRIAD database. The MIRIAD investigators did not participate in analysis or writing of this report. The MIRIAD dataset is made available through the support of the UK Alzheimer’s Society (Grant RF116). The original data collection was funded through an unrestricted educational grant from GlaxoSmithKline (Grant 6GKC). Data used in preparation of this article were obtained from the Alzheimers Disease Neuroimaging Initiative (ADNI) database (adni.loni.usc.edu). As such, the investigators within the ADNI contributed to the design and implementation of ADNI and/or provided data but did not participate in analysis or writing of this report. A complete listing of ADNI investigators can be found at: http://adni.loni.usc.edu/wp-content/uploads/how_to_apply/ADNI_Acknowledgement_List.pdf. Data collection and sharing for this project was funded by the 10.13039/100007333Alzheimer’s Disease Neuroimaging Initiative (ADNI) (National Institutes of Health Grant U01 AG024904) and DOD ADNI (Department of Defense award number W81XWH-12-2-0012). ADNI is funded by the 10.13039/100000049National Institute on Aging, the National Institute of Biomedical Imaging and Bioengineering, and through generous contributions from the following: AbbVie, Alzheimers Association; Alzheimers Drug Discovery Foundation; Araclon Biotech; BioClinica, Inc.; Biogen; Bristol-Myers Squibb Company; CereSpir, Inc.; Cogstate; Eisai Inc.; Elan Pharmaceuticals, Inc.; Eli Lilly and Company; EuroImmun; F. Hoffmann-La Roche Ltd and its affiliated company Genentech, Inc.; Fujirebio; GE Healthcare; IXICO Ltd.; Janssen Alzheimer Immunotherapy Research & Development, LLC.; Johnson & Johnson Pharmaceutical Research & Development LLC.; Lumosity; Lundbeck; Merck & Co., Inc.; Meso Scale Diagnostics, LLC.; NeuroRx Research; Neurotrack Technologies; Novartis Pharmaceuticals Corporation; Pfizer Inc.; Piramal Imaging; Servier; Takeda Pharmaceutical Company; and Transition Therapeutics. The Canadian Institutes of Health Research is providing funds to support ADNI clinical sites in Canada. Private sector contributions are facilitated by the Foundation for the National Institutes of Health (www.fnih.org). The grantee organization is the Northern California Institute for Research and Education, and the study is coordinated by the Alzheimers Therapeutic Research Institute at the University of Southern California. ADNI data are disseminated by the Laboratory for Neuro Imaging at the University of Southern California. Data were also provided in part by the Human Connectome Project, WU-Minn Consortium (Principal Investigators: David Van Essen and Kamil Ugurbil; 1U54MH091657) funded by the 16 NIH Institutes and Centers that support the 10.13039/100000135NIH Blueprint for Neuroscience Research; and by the McDonnell Center for Systems Neuroscience at Washington University.

## 5 Appendix

### 5.1 Dice score and Hausdorff metric compared between *CerebNet,* ACAPULCO^rt^, original ACAPULCO and SUIT+FS

**Fig. 8:**
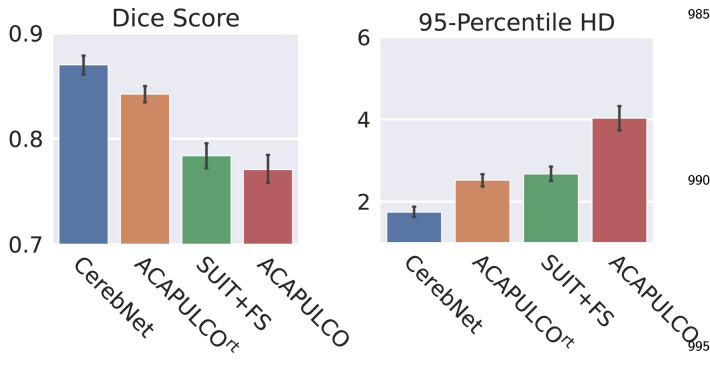
Comparison of Dice and Robust Hausdorff Distance (HD95) metrics for CerebNet, the retrained ACAPULCO^rt^, SUIT+FS as well as the original (published) ACAPULCO on our test set. Note, the direct comparison of *CerebNet* and the original ACAPULCO does not correct for the differences in the training datasets; ACAPULCO^rt^ corrects for this difference (see text).

To motivate our choice of pre-training with SUIT+FS and illustrate the impact of the dataset, we compare four methods on our test set: *CerebNet*, the ACAPULCO^rt^ (Han et al., 2020b) (both trained on our training set), as well as the original ACAPULCO (Han et al., 2020b) and SUIT+FS (Diedrichsen et al., 2009) (both trained on their individual datasets). We compare each prediction with our manually labeled reference segmentation to obtain average Dice and Robust Hausdorff metrics for each method in Fig. 8. Two observations are notable: 1. SUIT+FS outperforms the original ACAPULCO; 2. ACAPULCO^rt^ outperforms ACAPULCO (and SUIT+FS). Both results are expected and illustrate how much different labeling protocols (e.g. along the CGM/CWM border) can impact the performance and ranking of methods. These results confirm that inconsistent labeling protocols between training and test significantly impact the measured performance even to the level of contradicting previous rankings (Carass, Cuzzocreo, Han, Hernandez-Castillo, Rasser, Ganz, Beliveau, Dolz, Ben Ayed, Desrosiers, Thyreau, Romero, Coupé, Manjón, Fonov, Collins, Ying, Onyike, Crocetti, Landman, Mostofsky, Thompson, Prince, 2018, Han, Carass, He, Prince, 2020). Therefore, we 1. choose SUIT+FS for pre-training and 2. retrain ACAPULCO (yielding ACAPULCO^rt^) so that our methodological comparison are fair and not impacted by the choice of protocols.

This analysis also raises the question of how the performance of two pipelines may be compared fairly (a pipeline evaluation includes the impact of both the training dataset and the method). This is specifically difficult if protocols differ and higher quality reference standards are not available. While retraining on the same dataset yields a fair, direct comparison of methods (see for example section 3.2), pipeline comparisons, in situations where retraining is not feasible, require indirect evaluations based on segmentation-derived metrics instead of segmentation maps, e.g. whether and how well volume estimates can be used to differentiate between patient groups as done for SCA3 in Section 3.5.

### 5.2 Correlation of SARA sum scores with volumetric estimates

We perform a correlation analysis between the SARA sum score and regional volumes. Table 2 shows individual Kendell Tau coefficients for three methods: *CerebNet*, ACAPULCO (original) and ACAPULCO^rt^ (retrained). We also report whether the analysis achieved statistical significance. However, we would like to note that SCA3 is not a pure cerebllar disease like for example SCA6. The patterns of neurodegeneration in SCA3 include non-cerebellar structures, e.g. the basal ganglia or the peripheral nerve system. Progressive neurodegeneration of the cerebellum might be the main driver of ataxia severity in SCA3, but symptoms resulting from non-cerebellar manifestations, like e.g. spasticity or polyneuropathy have a direct impact on SARA items such as gait and stance. This should be taken into account in the interpretation of the correlation.

**Table 2:** Correlation of SARA sum score with each cerebellar volume for CerebNet, ACAPULCO and ACAPULCO^rt^. Kendell Tau correlation coefficients are given, and statistical significance of the correlation is indicated by * (p<.05) and ** (p<.01). For each volume, the most negative, statistically significant correlation coefficient is printed in boldface.

	CerebNet	ACAPULO	ACAPULCO^rt^
		(original)	(retrained)
Left I-IV	-0.204**	-0.134*	-0.211**
Right I-IV	-0.235**	-0.167*	-0.208**
Left V	-0.0602	-0.213**	-0.0593
Right V	-0.00294	-0.194**	-0.0401
Left VI	-0.306**	-0.227**	-0.284**
Vermis VI	-0.0954	-0.0197	-0.0874
Right VI	-0.322**	-0.225**	-0.264**
L. Crus I	-0.252**	-0.287**	-0.214**
R. Crus I	-0.200**	-0.199**	-0.151*
L. Crus II	-0.219**	-0.267**	-0.145*
R. Crus II	-0.214**	-0.179**	-0.154*
Left VIIb	-0.385**	-0.241**	-0.341**
Right VIIb	-0.410**	-0.364**	-0.314**
Vermis VII	-0.144*	-0.139*	-0.0765
Left VIIIa	-0.299**	-0.113	-0.264**
Right VIIIa	-0.315**	-0.100	-0.408**
Left VIIIb	-0.317**	-0.311**	-0.374**
Right VIIIb	-0.254**	-0.344**	-0.323**
Vermis VIII	-0.182**	-0.0941	-0.170**
Left IX	-0.258**	-0.159*	-0.232**
Vermis IX	-0.0650	-0.0327	-0.0760
Right IX	-0.292**	-0.248**	-0.240**
Left X	-0.265**	0.0101	-0.207**
Vermis X	-0.115	-0.0667	-0.0270
Right X	-0.298**	-0.0598	-0.253**
CWM	-0.583**	-0.440**	-0.549**

### 5.3 CerebNet segmentation in sporadic and hereditary ataxias

**Fig. 9:**
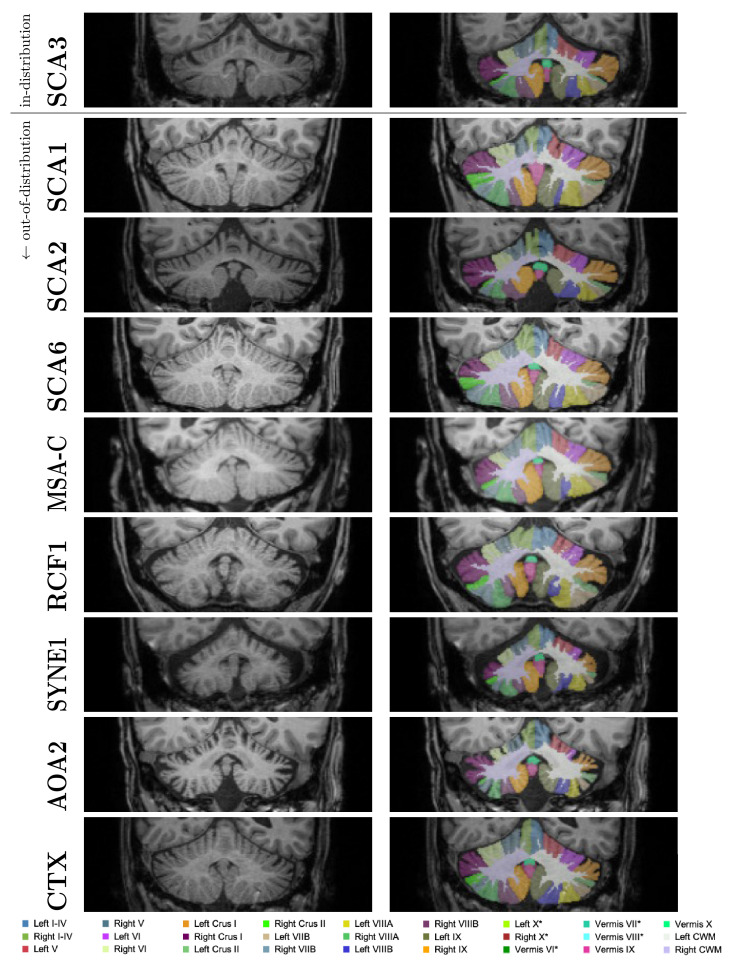
Qualitative “out-of-distribution” evaluation of *CerebNet*: Segmentation maps for pathologies, which are not part of the training (SCA1, SCA2, SCA6, MSA-C, RFC1, SYNE1, AOA2 and CTX) together with an in-distribution example (SCA3). Images illustrated here are randomly picked from a larger repository of images and represent average performance. *: Label is not visible in the shown slice.

To illustrate the robustness of *CerebNet* to “out-of-distribution” samples, we show some qualitative examples of segmentations in Fig. 9. While these results indicate good performance across many pathologies, studies utilizing *CerebNet* to segment patients with these or other diseases should still ensure the performance also translates to their datasets by performing a formal validation, or, at least, rigorous quality assurance as laid out in the Discussion.

## Data and Code Availability Statement

The MRI data is not publicly available because of data protection regulations. Access can be provided upon reasonable request to scientists in accordance with our Data Use and Access Policy. Requests to access the data should be directed to Jennifer Faber at Jennifer.Faber@dzne.de.

The source code of CerebNet will be made publicly available on Github (https://github.com/Deep-MI/FastSurfer) upon acceptance.

## Footnotes

3 Excluding cases from the OASIS-1 reliability section.

4 Since the ACAPULCO training dataset is not available publicly, we cannot retrain *CerebNet* with this data.
